# Vagus Nerve Stimulation as a Treatment for Fear and Anxiety in Individuals with Autism Spectrum Disorder

**DOI:** 10.20900/jpbs.20220007

**Published:** 2022-09-23

**Authors:** Tanushree Shivaswamy, Rimenez R. Souza, Crystal T. Engineer, Christa K. McIntyre

**Affiliations:** Texas Biomedical Device Center and School of Behavioral and Brain Sciences. The University of Texas at Dallas, 800 West Campbell Road, Richardson, TX 75080, USA

**Keywords:** behavioral therapy, exposure therapy, neurodevelopmental disorders, neuromodulation, rehabilitation

## Abstract

Anxiety disorders affect a large percentage of individuals who have an autism spectrum disorder (ASD). In children with ASD, excessive anxiety is also linked to gastrointestinal problems, self-injurious behaviors, and depressive symptoms. Exposure-based cognitive behavioral therapies are effective treatments for anxiety disorders in children with ASD, but high relapse rates indicate the need for additional treatment strategies. This perspective discusses evidence from preclinical research, which indicates that vagus nerve stimulation (VNS) paired with exposure to fear-provoking stimuli and situations could offer benefits as an adjuvant treatment for anxiety disorders that coexist with ASD. Vagus nerve stimulation is approved for use in the treatment of epilepsy, depression, and more recently as an adjuvant in rehabilitative training following stroke. In preclinical models, VNS shows promise in simultaneously enhancing consolidation of extinction memories and reducing anxiety. In this review, we will present potential mechanisms by which VNS could treat fear and anxiety in ASD. We also discuss potential uses of VNS to treat depression and epilepsy in the context of ASD, and noninvasive methods to stimulate the vagus nerve.

## INTRODUCTION

Autism spectrum disorder (ASD) is a neurodevelopmental disorder that affects 1 in 44 children [1]. The common diagnostic criteria for disorders that fall into the spectrum of ASD are persistent impairments in social interaction and the presence of restricted, repetitive patterns of behaviors, interests, or activities [2]. Other impairments are often overlooked, such as heightened fear responses [3], and anxiety [4]. These symptoms can lead to maladaptive behaviors such as tantrums, aggression, and self-injurious behaviors [5]. In addition, forty percent of individuals with ASD are co-diagnosed with anxiety disorders [6], which include phobias, social anxiety disorder, and obsessive-compulsive disorder [7]. Specific phobias affect 30% of individuals with ASD [4]. Individuals with ASD may also be at higher risk for trauma-related disorders such as posttraumatic stress disorder (PTSD) [8].

Cognitive behavioral therapies (CBTs) have proven to be effective methods to treat anxiety disorders in patients with ASD [9,10]. They are designed to rehabilitate behavior through experience-based learning and brain plasticity [11,12]. Exposure is the most applied component of CBTs to address abnormal fear and anxiety [13], and it is commonly used to treat PTSD and phobias [14,15]. These therapies reinforce appropriate behavior via repeated exposures to fear and anxiety triggers in a safe and controlled situation. They are dependent upon extinction learning [16], or the process of extinguishing maladaptive responses through newly learned associations that compete with the fear memory [17,18]. Exposure-based therapies are over three times less effective in treating phobias in patients with ASD than in other populations [19]. Communication deficits and difficulties with emotional cognition may interfere with progress in therapy [4]. Generalization, or the ability to apply what is learned in one context across various situations, is also impaired in individuals with ASD [20]. Moreover, high relapse rates and low tolerability of CBTs among patients with ASD suggest that therapy improvements and other strategies are needed [21,22]. Thus, augmentation could be a potential strategy to improve outcomes of CBTs in patients with ASD who have anxiety disorders.

This perspective discusses evidence suggesting that vagus nerve stimulation (VNS) paired with CBTs may offer benefits for anxiety disorders that coexist with ASD. Studies in rodents and humans have suggested that VNS can be a promising strategy to reduce anxiety [23–26], and enhance learning and memory [27–29] and fear extinction [30–32]. Preclinical studies also indicate that VNS promotes generalization of fear extinction in rodents [33,34]. Electrical stimulation of the cervical vagus nerve is an FDA-approved treatment for drug-resistant epilepsy and depression [35]. Based on results of human trials [36], VNS was recently approved as a rehabilitation system to treat upper extremity motor deficits associated with stroke, and preclinical studies suggest that it also has the potential for use as an adjuvant to improve outcomes of speech therapies [37]. Despite the strong evidence that VNS can improve recovery in several conditions, studies have not been conducted on its potential to improve therapies for treating fear and anxiety in individuals with ASD.

## EXPOSURE-BASED THERAPIES IN ASD

Traditional and virtual forms of exposure therapy are commonly used to treat phobias in ASD. In vivo exposure therapy is a form of traditional exposure therapy in which patients are directly exposed to the object or situation that they fear [38]. This form of exposure therapy has proven effective in randomized controlled trials of patients with ASD who have anxiety disorders [9,10]. It is not always practical to return to the site of the trauma, so imaginal exposure therapy can be used in the therapist’s office. Imaginal exposure therapy, wherein patients are exposed to their own thoughts and mental images, benefitted young people with ASD who had obsessive-compulsive disorder to a lesser degree than typically developing children [39]. A central part of in vivo and imaginal exposure therapy is homework, where patients are asked to reinforce what they have learned in therapy outside of the clinical setting [40]. However, homework compliance is low in patients with ASD who receive exposure-based therapy for obsessive-compulsive disorder [39]. Additionally, responses to reinforcers presented in traditional exposure therapy may be unpredictable due to differences in verbal and intellectual abilities among individuals with ASD [20].

Virtual reality (VR) is used to enhance extinction learning [41]. Virtual reality has been successfully used in CBTs for specific phobias in ASD, such as train/bus travel, social situations, and street crossing [19]. One benefit to using VR is that it can incorporate more triggers that could provoke a fear response within a given situation [41]. Also, the therapist has control over all aspects of the virtual environment and can customize and adjust based on the patients’ needs [41]. Additionally, VR can be beneficial for phobias where simulation of certain aspects of the feared situation would be impractical using traditional methods [42]. However, VR may induce simulator-related side-effects such as dizziness and vertigo [42]. Despite this limitation, VR and other strategies that could enhance the efficacy and practicality of exposure-based therapies may be important allies in improving outcomes of CBTs in individuals with ASD.

## VNS AS AN ADJUVANT TO EXPOSURE THERAPY

The vagus nerve is the tenth cranial nerve, and it is the longest and most widely distributed nerve in the nervous system [43]. Direct stimulation of the vagus nerve is performed by surgically implanting a cuff electrode on the left vagus nerve at the cervical level [44]. In humans and rodents, vagus nerve stimulation (VNS) enhances performance on cognitive tasks. Early studies demonstrated VNS-induced enhancement of long-term avoidance memory in rats [27,28] and recognition memory in humans [45]. Pairing VNS with a visual discrimination task enhances reversal learning, a form of cognitive flexibility, in rats [29]. Additionally, VNS accelerates extinction learning and decreases multiple physiological measures of fear expression in rodents exposed to an inhibitory avoidance task [46]. Accelerating treatment and enhancing cognition could benefit patients with ASD who may be slow to acquire new information during exposure-based therapy. Given that VNS enhances multiple forms of learning, pairing it with exposure-based therapies could make them more efficient for patients with ASD.

Pre-clinical studies in rodents show that VNS enhances and accelerates extinction of conditioned fear when it is paired with extinction training [30]. Furthermore, recent studies indicate that VNS reverses fear extinction impairments in rat models for PTSD [31,32]. Studies in rodents also indicate that VNS promotes generalization of extinction memory [33,34]. For example, VNS paired with extinction of conditioned fear of an auditory stimulus led to generalization of extinction of fear of an auditory stimulus that was co-conditioned during fear acquisition but not present during extinction training [34]. In another study, VNS enhanced extinction of olfactory conditioned fear, and pairing VNS with extinction of an auditory cue led to reduced conditioned fear of the olfactory cue, and vice versa [33]. By promoting generalization of extinction across sensory modalities, VNS could make exposure-based therapies more effective for patients with ASD and reduce relapse.

Pairing VNS with extinction training can simultaneously enhance extinction of conditioned fear and attenuate elevated anxiety, hyperarousal, and social withdrawal in rodent models for PTSD [31,32]. The single-prolonged stress (SPS) model, in which rats are exposed to a single session of prolonged stress that includes restraint followed by a forced swim, is commonly used to model PTSD, and SPS treatment leads to impaired retention of extinction memory [47]. Noble and colleagues (2017) found that SPS-treated rats showed elevated anxiety-like behavior in an elevated plus maze, increased acoustic startle responses, and increased marble burying, suggesting that SPS treatment increased anxiety, arousal, and avoidance. Administration of VNS during extinction training reversed SPS effects on anxiety, arousal, and avoidance [31]. In a follow up study, rats were exposed to SPS and protracted aversive conditioning to produce stronger conditioned fear that more closely resembles PTSD-like trauma. Once again, administration of VNS during extinction training attenuated anxiety-like behavior measured on the elevated plus maze, however, renewal of the conditioned fear brought about a return of anxiety in these animals [32].

The studies described above used 30-s-long trains of VNS at an intensity of 0.4 mA, and 100–500 microsecond pulse widths, to enhance extinction of conditioned fear when VNS overlapped with a 30-s presentation of an auditory conditioned stimulus during extinction training. However, recent studies have indicated that effective VNS parameters for enhancing extinction range from 0.4 mA to 0.8 mA and from short bursts (0.5 s) to long trains (30 s) when VNS overlaps with presentation of the conditioned stimulus [30–34,48]. In addition, a recent study showed that short VNS bursts (0.5 s) at an intensity of 0.8 mA paired with conditioned stimulus presentations enhanced extinction of conditioned fear in rats and prevented fear renewal and reinstatement in the rat model for PTSD [48]. Observations of VNS effects on memory consolidation and reversal learning indicate that stimulation could produce an inverted-U response with increasing intensity [27,28,45] or frequency [29] and a non-monotonic function for VNS effects on cortical plasticity, where an intensity of 0.8 mA is effective while higher intensities are not as effective at promoting changes in auditory [49] and motor cortex [50]. A similar relationship has been observed for VNS effects on extinction of conditioned fear in a model of PTSD [33]. These findings suggest that a narrow range of stimulation intensities enhances plasticity and recovery when VNS is paired with training. Stimulation pulse width and frequency should be carefully considered because they can interact with intensity to contribute to the total charge delivered [29,51].

## POTENTIAL MECHANISMS BY WHICH VNS COULD TREAT FEAR AND ANXIETY

### Neuromodulators

The mechanism by which VNS could reverse extinction impairments in rodent models of ASD may be increased plasticity in the pathway from the prefrontal cortex to the amygdala. Vagus nerve stimulation increases norepinephrine (NE) in the basolateral amygdala [52], prefrontal cortex [53], and hippocampus [54]. Norepinephrine in these brain regions is necessary for consolidation of emotionally arousing memories [55,56]. Although VNS effects on extinction appear to be mediated, at least partly, by increasing NE in the prefrontal cortex, the precise timing of VNS paired with exposure may increase the signal to attend to the presented cues, or to consolidate the extinction memory. Synergistic tonic and phasic firing of the locus coeruleus (LC) is required to increase alertness and attention in states of hyperarousal [57]. Vagus nerve stimulation increases phasic LC activity [58]. Therefore, the mechanism by which VNS could increase attention to the conditioned cues presented during extinction training in rodent models of ASD may be increased transient firing of the LC.

### Neural Plasticity

Vagus nerve stimulation modulates plasticity in the pathway from the infralimbic prefrontal cortex to the basolateral amygdala, a circuit which is critically involved in extinction learning [59]. Beyond its potential to increase plasticity in extinction networks, preclinical studies show that VNS also modulates plasticity in other regions of the cortex. In noise-exposed rats, pairing VNS with tones eliminated the physiological and behavioral correlates of tinnitus, which lasted for weeks after the end of therapy [60]. Furthermore, pairing VNS with tones increases plasticity across the rodent auditory cortex [61]. Additionally, moderate intensity VNS enhances plasticity in the rodent motor cortex when paired with forelimb movements [50]. Norepinephrine released by the locus coeruleus is involved in many forms of plasticity, including hippocampal long-term potentiation [62,63], long-term depression [64–66], and blocking depotentiation [67]. In addition, locus coeruleus projections to the dorsal raphe nucleus slightly increase the release of serotonin [68]. Norepinephrine and serotonin are both required for VNS to facilitate cortical plasticity [69]. Therefore, precise pairing of VNS with training or rehabilitation could be a way of promoting learning-related plasticity in rodent models of neurodevelopmental disorders, and patients with ASD.

### Anxiolytic Effects

Chronic VNS reduces anxiety in rats [25,70] and in humans [24], and recent studies indicate that acute VNS can have an immediate anxiolytic effect in rats. Vagus nerve stimulation administration before testing reduced anxiety-like behavior in naïve rats in the elevated plus maze and novelty suppressed feeding test [26]. The vagus nerve is called the “vagal brake” because it is part of the parasympathetic branch of the peripheral nervous system, and it counteracts the sympathetic nervous system-mediated effects of stress on the heart and other organs [71]. The role of the parasympathetic nervous system in VNS effects on anxiety and extinction enhancement was tested in rodents using the cholinergic receptor antagonist methyl scopolamine, which blocks muscarinic receptors in the peripheral nervous system but does not enter the brain [23]. Noble and colleagues found that the immediate VNS-dependent reduction in anxiety-like behavior on the elevated plus maze depended on signaling at peripheral muscarinic receptors, but VNS enhancement of extinction did not. Therefore, the anxiolytic effects of VNS do not appear to be necessary for VNS enhancement of extinction. These findings suggest that VNS could reduce the sympathetic stress response in ASD by increasing parasympathetic nervous system signaling at peripheral cholinergic receptors and thus, in addition to enhancing extinction of learned fears, VNS can reduce conditioned stress responses in the minutes after it is administered.

Individuals with ASD, particularly those with intellectual disabilities, are likely to drop out of therapy because they may not tolerate some aspects of the therapy, such as uncertainty [22]. By reducing the sympathetic stress response, VNS could make exposure-based therapies more tolerable for patients with ASD and reduce dropout. Extinction enhancement, learning acceleration, and anxiety reduction make VNS a promising adjuvant to exposure therapy because it may simultaneously make therapy more effective, more efficient, and more tolerable for patients with ASD.

### Anxiety in Animal Models of ASD

Autism spectrum disorder is an idiopathic condition, but preclinical and clinical research indicates that a variety of genetic and environmental factors could increase the risk of this disorder [72]. Some genetic rat models of ASD, such as *Arid1b* knockout, *Chd8* knockout, *Fmr1* knockout, and *Scn2a* knockout, exhibit elevated anxiety, impaired fear learning, and impaired fear extinction [73–76]. In addition, environmental factors, such as prenatal maternal stress [77], and prenatal and postnatal exposure to toxins [78], could increase the risk of ASD. For instance, prenatal exposure to the teratogen valproic acid (VPA) is associated with increased risk of ASD [79,80]. Valproic acid is a gamma aminobutyric acid agonist and histone deacetylase inhibitor [81,82] that is used as a treatment for epilepsy [83]. Studies on the use of VPA in rodent models of ASD reveal that in utero exposure to VPA on the twelfth gestational day produces elevated anxiety and impaired fear extinction, among other ASD-like phenotypes and neural abnormalities [84–86]. Vagus nerve stimulation may be used to attempt to reverse extinction impairments and heightened anxiety in rats who are prenatally exposed to VPA as a preclinical test of the hypothesis that VNS can overcome ASD-related barriers for treatment of anxiety disorders.

### Chronic VNS

Surgical implantation of a VNS electrode is invasive, and it is, therefore, not an ideal first line of adjuvant therapy. However, beyond the evidence that acute VNS may be a beneficial adjuvant to exposure-based therapies, chronic VNS may also provide benefits in treatment of other ASD comorbidities such as epilepsy and depression. Therefore, surgical implantation of the electrode may be practical for some individuals with ASD.

### Epilepsy

Epilepsy affects approximately 25% of individuals with ASD [87], and individuals with epilepsy are 20% more likely to have an autism diagnosis [88]. In treatment of epilepsy, VNS improves quality of life and increases alertness, communication, independence, memory, mood, and sleep [45,89,90]. Studies on the effects of chronic VNS in patients with ASD find an improvement in seizures, in mood, alertness, and social functioning [91–93], and a decrease in aggression [94], which could be associated with improved emotion regulation abilities [95]. However, decreases in aggression are not associated with or explained by a lower seizure frequency. Because VNS promotes pairing-specific plasticity [96], acute VNS paired with cognitive behavioral therapy may provide benefits in treating anxiety in patients with ASD who use chronic VNS to reduce epileptic seizures.

### Depression

Depression affects 53% of individuals with ASD and is characterized by anhedonia, which presents as a loss of interest in rewarding activities, refusal to attend structured activities, and decreased responses to stimuli that were previously motivating [97]. Individuals with ASD who have limited verbal skills and intellectual disabilities can present with symptoms of depression such as self-injurious or destructive behavior, or increased social withdrawal [98]. Symptoms of depression are associated with decreased cortisol responses to stress, which could increase the risk of unhealthy dysregulation of the hypothalamus-pituitary-adrenal (HPA) axis [99]. Some studies show that VNS can attenuate dysfunction in the HPA axis [100,101]. Moreover, a five-year study demonstrated that patients who were given VNS as a treatment for depression showed better clinical responses than patients in the treatment-as-usual group, including a higher cumulative response rate [102]. Therefore, an implanted VNS device may provide effective treatment for ASD patients with medication-resistant depression, and VNS paired with cognitive behavioral therapy could provide additional benefits in treating fear and anxiety.

## LIMITATIONS AND FUTURE DIRECTIONS FOR VNS

The method that is most used to stimulate the vagus nerve in animals and humans utilizes the surgical implantation of a cuff electrode on the left vagus nerve at the cervical level [44,103]. This method of VNS administration is FDA approved. Most preclinical studies where VNS is paired with therapy utilize this method of stimulating the vagus nerve. Surgery and a chronic implant are limitations of this approach. Parents may be especially hesitant to request surgical implantation of a device in their children. However, VNS is a safe and effective treatment for patients with ASD who have epilepsy [91–93]. Future preclinical research should be aimed at identifying the mechanisms by which VNS could treat fear and anxiety in ASD so less invasive approaches can be designed to target those pathways.

## LESS INVASIVE VNS

Less invasive approaches to stimulating the vagus nerve could circumvent problems associated with the surgery and chronic implant. Two less invasive methods are the ReStore system and transcutaneous VNS. The ReStore system utilizes a miniature wireless pulse generator that is implanted on the vagus nerve. It can be programed wirelessly and has no battery or leads [104]. The ReStore system has received FDA approval for an early safety and feasibility study (Kilgard, M.P. (2021) *Targeted Plasticity Therapy for Upper Limb Rehabilitation in Spinal Cord Injuries*. Identification No. NCT04288245. Retrieved from https://clinicaltrials.gov/ct2/show/NCT04288245). Although results are promising and this is an advance to the current state of VNS, surgical implantation is still needed to install the device.

Transcutaneous VNS utilizes a device that is placed on the concha of the left ear to stimulate the auricular branch of the vagus nerve [105]. This noninvasive approach has been proposed as a treatment for ASD because it regulates neuroimmune responses and produces therapeutic effects for comorbid disorders of ASD such as epilepsy and depression [106]. For example, tVNS reduces symptoms of depression while increasing the resting-state functional connectivity between the amygdala and dorsolateral prefrontal cortex [107]. However, studies using tVNS paired with extinction in tests of anxiety and fear memory in humans have yielded mixed results [108,109]. Thus, future research should be aimed at optimizing tVNS to increase the tolerability and efficacy of exposure-based therapies in patients with ASD.

## CONCLUSIONS

Pairing VNS with exposure-based and rehabilitative therapy might enhance outcomes for individuals with anxiety disorders. Future research on invasive and noninvasive VNS should be aimed at identifying optimal stimulation parameters to make it a safe and effective adjuvant to cognitive behavioral therapies and other rehabilitative therapies for patients with ASD. Side-effects of VNS include voice alterations and hoarseness, which can be alleviated by adjusting VNS parameters [110]. Compared to the long trains of VNS that are utilized in treating human epilepsy, a decreased amplitude and duration are effective parameters for VNS to enhance extinction and pairing-specific plasticity in rodents [48,51]. Vagus nerve stimulation might be an attractive adjuvant to exposure-based therapies for patients with ASD who have severe anxiety and for such individuals who already have an implant for epilepsy or depression.

## Data Availability

No data were generated from the study.
